# Relation Between Plasma Proteomics Analysis and Major Adverse Cardiovascular Events in Patients With Stable Coronary Artery Disease

**DOI:** 10.3389/fcvm.2022.731325

**Published:** 2022-02-08

**Authors:** Mihaela Ioana Dregoesc, Adrian Bogdan Ţigu, Siroon Bekkering, Charlotte D. C. C. van der Heijden, Sorana Daniela Bolboacǎ, Leo A. B. Joosten, Frank L. J. Visseren, Mihai G. Netea, Niels P. Riksen, Adrian Corneliu Iancu

**Affiliations:** ^1^Department of Cardiology, “Iuliu Haţieganu” University of Medicine and Pharmacy, Cluj-Napoca, Romania; ^2^Medfuture—The Research Center for Advanced Medicine, “Iuliu Haţieganu” University of Medicine and Pharmacy, Cluj-Napoca, Romania; ^3^Murdoch Children's Research Institute, The Royal Children's Hospital, Parkville, VIC, Australia; ^4^Department of Internal Medicine, Radboud University Medical Center, Radboud Institute for Molecular Life Sciences, Nijmegen, Netherlands; ^5^Department of Medical Informatics and Biostatistics, “Iuliu Haţieganu” University of Medicine and Pharmacy, Cluj-Napoca, Romania; ^6^Department of Vascular Medicine, University Medical Center Utrecht, Utrecht, Netherlands; ^7^Department of Immunology and Metabolism, Life and Medical Sciences Institute, University of Bonn, Bonn, Germany

**Keywords:** inflammation, biomarkers, proteomics, atherosclerosis, coronary artery disease

## Abstract

**Objective:**

Despite the advances in the control of traditional risk factors, coronary artery disease (CAD) remains the greatest cause of morbidity and mortality. Our aim was to establish the relation between plasma proteomics analysis and the risk of cardiovascular events in patients with stable CAD.

**Materials and Methods:**

Patients with stable CAD and documented coronary atherosclerosis were screened for inclusion. Using proximity extension assays, 177 plasma proteins were simultaneously measured. The endpoint consisted of the first major adverse cardiovascular event (MACE) and was the composite of cardiovascular death, acute coronary syndrome, stroke, transient ischemic attack, or acute limb ischemia at 18 months follow-up. Cox proportional-hazards regression with adjustment for multiple comparisons was used to identify biomarkers for the outcomes of interest.

**Results:**

The cohort consisted of 229 patients. Six mediators were associated with MACE (*p* < 0.001). For these markers, the risk of MACE was calculated: tumor necrosis factor receptor superfamily member 13B (HR = 1.65; 95% CI: 1.30–2.10), C-C motif chemokine-3 (HR = 1.57; 95% CI: 1.23–1.98), decorin (HR = 1.65; 95% CI: 1.26–2.16), fibroblast growth factor-23 (HR = 1.56; 95% CI: 1.23–1.99), tumor necrosis factor-related apoptosis-inducing ligand-receptor 2 (TRAIL-R2) (HR = 1.61; 95% CI: 1.23–2.11), and tumor necrosis factor receptor superfamily member 10A (HR = 1.69; 95% CI: 1.25–2.29). Except for TRAIL-R2, the other proteins were associated with MACE independent of age, sex, diabetes mellitus, or estimated glomerular filtration rate.

**Conclusions:**

In patients with stable CAD, five novel biomarkers were identified as independent risk factors for adverse outcomes. Novel biomarkers could represent pharmacological targets for the prevention of adverse cardiovascular events.

## Introduction

Despite the advances in the control of traditional risk factors for cardiovascular disease (CVD), coronary artery disease (CAD) remains the greatest cause of morbidity and mortality (1).

Atherosclerosis is considered as an inflammatory disease of the arterial wall. Circulating markers of inflammation, such as high-sensitivity C-reactive protein (hs-CRP) and interleukin-6 (IL-6), correlate with disease activity and with adverse outcomes following acute events (1, 2). The inflammatory hypothesis of atherothrombosis was tested in the randomized, large-scale CANTOS trial, which showed that targeting interleukin-1β (IL-1β) with canakinumab reduced cardiovascular event rates in acute myocardial infarction survivors (3). The nonspecific anti-inflammatory drug colchicine also lowered the risk of ischemic cardiovascular events in the recent COLCOT and LoDoCo2 trials (4, 5).

The widely accepted risk stratification models, such as the Framingham risk score, only have a modest predictive value for the development of adverse events (6–10). This emphasizes the need for novel and better biomarkers predictive of atherosclerosis progression and plaque destabilization. Technical advances have enabled simultaneous measurement of large numbers of plasma proteins and have facilitated the interpretation of large data sets. Two studies have recently demonstrated the superiority of proteome-based models vs. classical risk factors models in predicting the presence of high-risk atherosclerotic plaques (10) or the risk of adverse cardiovascular events in the general population (11). However, supporting data remains limited regarding the use of proteomics to identify patients with stable coronary artery disease at risk for future adverse outcomes.

In this context, we set out to perform a prospective study in a cohort of patients with stable CAD and angiographically documented coronary atherosclerosis. We performed targeted proteomics through the simultaneous measurement of 177 circulating inflammatory proteins and investigated their relationship with a first future cardiovascular adverse event.

## Materials and Methods

This was a prospective, observational, single-center cohort study. Between May 2017 and September 2018, 1,020 consecutive patients with symptomatic stable CAD were screened for inclusion. Criteria for inclusion were at least one coronary atherosclerotic stenosis above 30% on angiography, and inducible myocardial ischemia at the treadmill or imaging stress testing. The exclusion criteria consisted of any documented past acute coronary syndrome, stroke, transient ischemic attack, or acute limb ischemia.

The research protocol was approved by the Institution's Ethics Committee on research on humans, and all patients gave written informed consent. All the procedures followed were in accordance with institutional guidelines. The study protocol conforms to the 1975 Declaration of Helsinki.

The classic cardiovascular risk factors were recorded in all patients. An active smoker was defined as a person who has smoked more than 100 cigarettes during their lifetime and has smoked in the last month. Arterial hypertension was defined as systolic blood pressure >140 mmHg or diastolic blood pressure >90 mmHg, according to the current practice guidelines (12). The definition of diabetes mellitus was based on the 2006/2011 World Health Organization recommendations (13, 14). The estimated glomerular filtration rate (eGFR) was calculated based on the Modification of Diet in Renal Disease Study (MDRD) formula.

To evaluate the extent of coronary atherosclerosis, SYNTAX Score I was calculated by a senior interventional cardiologist based on the most recent coronary angiography (http://syntaxscore.org/calculator/syntaxscore/frameset.htm).

### Blood Sampling

Total cholesterol, high-density lipoprotein (HDL) cholesterol, and triglycerides were measured in fasting plasma using a Roche cobas c501 chemistry analyzer. Low-density lipoprotein (LDL) cholesterol was calculated with the Friedewald formula. Total blood cell counts were determined with an automated Sysmex-XN 1000 hematology analyzer (Sysmex, Hamburg, Germany).

### Proteome Analysis by Proximity Extension Assays Technology

All EDTA plasma samples were shipped to Olink Proteomics AB (Uppsala, Sweden) for analysis. Using the previously described proximity extension assays (PEA) technology (15), the levels of 177 inflammatory proteins from the Olink Cardiovascular II and Inflammatory panels were measured (Supplementary Table 1). In brief, oligonucleotide-labeled antibody probe pairs bind to their respective targeted protein in each sample, and if the two probes are brought in close proximity the oligonucleotides will hybridize in a pair-wise manner. Data is quality controlled and normalized using an internal extension control and an inter-plate control, to adjust for intra- and inter-run variation (https://www.olink.com/resources-support/document-download-center/; accessed August 2020). The final data is presented in Normalized Protein eXpression (NPX) values, which is an arbitrary unit on a log2-scale. High NPX values correspond to higher protein concentrations. One protein was excluded from the analysis because >20% of individual measurements were below the lower limit of detection.

### Follow-Up

Patient follow-up was performed after 18 months. The endpoint consisted of the first major adverse cardiovascular event (MACE) and was the composite of cardiovascular death, acute coronary syndrome, ischemic stroke, transient ischemic attack, or acute limb ischemia.

### Statistical Analysis

Data distribution was assessed using Kolmogorov–Smirnov and D'Agostino tests. Quantitative continuous data were summarized as mean ± standard deviation (SD) whenever data followed the normal distribution; otherwise, median and interquartile range (Q1–Q3, where Q1 = first quartile and Q3 = third quartile) were used.

Continuous variables were compared with the Student *t*-test or Mann–Whitney test, and correlations were assessed using Spearman's coefficient, with the associated statistical test. Categorical data was presented as counts and proportions and compared with Chi-square or Fisher's exact tests.

Identification of biomarkers for the outcomes of interest was made in univariate Cox proportional-hazards regression, using a Benjamini–Hochberg adjusted significance level (16), at a controlled false discovery rate (FDR) of 2.5% (*p* < 0.001). To directly compare the risk for adverse outcomes, hazard ratios (HR) per 1 standard deviation (SD) increase in protein levels were computed, together with their associated 95% confidence intervals (95% CI). Multivariate Cox proportional-hazards regression analysis was applied to identify proteomic biomarkers independently associated with outcomes. All proteomic biomarkers that were statistically significant in the univariate analysis, at a controlled FDR of 2.5%, together with specific confounders for adverse outcomes (age, sex, diabetes mellitus, and estimated glomerular filtration rate, LDL-cholesterol and hypertension) were included in the multivariable models. Single imputation methods were used to reduce missing covariate data for estimated glomerular filtration rate (eGFR) (*n* = 5; 2.1%), TNFRSF10A (*n* = 1; 0.4%), TNFRSF13B (*n* = 1; 0.4%), DCN (*n* = 1; 0.4%), and TRAIL-R2 (*n* = 1; 0.4%), since incomplete case analysis leads to loss of statistical power and possible bias.

The statistical power analysis for the identified proteins as factors associated with MACE was retrospectively conducted considering the sample size, the values of observed MACE, and the desired significance level (α = 5%) for a two-tailed test. The *post-hoc* power analysis was made with G^*^Power (v. 3.1.9.2, © 1992–2014).

Statistical analysis was performed with GraphPad Prism version 8.4.2 for Windows (GraphPad Software, CA, USA) and MedCalc (v 10.3.0.0, MedCalc Software, Ostend, Belgium). A two-sided *p* < 0.05 was considered statistically significant.

## Results

### Baseline Characteristics

A cohort of 229 patients with a mean age of 64.9 years was recruited (Table 1). Men represented two thirds of the population. The vast majority of patients had hypertension, while more than a third of patients had type 2 diabetes mellitus. More than 90% of the patients were treated with statin therapy.

**Table 1:** Baseline patient characteristics in the study cohort.

**Parameter**	**Cohort (*n* = 229)**
**Clinical data**
Age (years)	64.9 ± 8.7
Males, *n* (%)	157 (68.5)
BMI (kg/m^2^)	29.2 ± 3.7
Waist (cm)	102 ± 12
Smokers, *n* (%)	20 (8.7)
Heart rate (beats/min), median (Q1–Q3)	68 (60–75)
Hypertension, *n* (%)	206 (90)
Systolic blood pressure (mmHg)	138 ± 22
Diastolic blood pressure (mmHg)	80 ± 11
Diabetes, *n* (%)	81 (35)
**Laboratory data**
Glycemia (mg/dl), median (Q1–Q3)	108.1 (97.0–132.3)
Total cholesterol (mg/dl)	168.0 ± 47.3
LDL-cholesterol (mg/dl)	91.2 ± 36.8
HDL-cholesterol (mg/dl)	42.8 ± 13.6
Triglycerides (mg/dl), median (Q1–Q3)	143.6 (100.6–199.7)
Creatinine (mg/dl), median (Q1–Q3)	0.9 (0.8–1.1)
eGFR (ml/min/1.73m^2^), median (Q1–Q3)	82.5 (68.9–99.3)
**Echocardiographic data**
LVEF (%)	53.0 ± 9.2
**Concomitant medication**
Aspirin, *n* (%)	191 (83)
P2Y12 inhibitor, *n* (%)	139 (61)
Anticoagulant (AVK/NOAC)	29 (13)
Betablocker, *n* (%)	188 (82)
ACE inhibitor/ARB, *n* (%)	175 (76)
Calcium channel blocker, *n* (%)	69 (30)
Nitrate, *n* (%)	78 (34)
Statin, *n* (%)	209 (91)
Fibrate, *n* (%)	16 (7)
**Atherosclerosis severity**
SYNTAX Score I, median (Q1–Q3)	11 (7–20.5)
SYNTAX Score II—PCI, median (Q1–Q3)	29.5 (22.8–35.7)
SYNTAX Score II—CABG, median (Q1–Q3)	26.7 (20.2–34.2)
**Revascularization**
Complete, *n* (%)	71 (31)
Incomplete, *n* (%)	80 (35)
Absent, *n* (%)	78 (34)

*Values are presented as mean ± SD, unless otherwise stated*.

*ACE, angiotensin converting enzyme; ARB, angiotensin receptor blocker; AVK, antivitamin K; BMI, body mass index; CABG, coronary artery bypass graft; eGFR, estimated glomerular filtration rate; HDL-cholesterol, high density lipoprotein cholesterol; LDL-cholesterol, low density lipoprotein cholesterol; LVEF, left ventricular ejection fraction; n, number; NOAC, novel oral anticoagulant; PCI, percutaneous coronary intervention; Q1, 1st quartile; Q3, 3rd quartile*.

At 18-months follow-up, 16% of patients reached the composite endpoint (Supplementary Table 2).

Patients who reached the composite endpoint were older (*p* = 0.02), had a higher body mass index (BMI) (*p* = 0.04), and lower eGFR (*p* = 0.01). In patients who reached the composite endpoint, coronary atherosclerosis at baseline was more extensive and complex, as evaluated by SYNTAX Score I (*p* = 0.04) and SYNTAX Score II – PCI (*p* = 0.006). The complete characteristics of the two groups are provided in Table 2. Univariate Cox proportional hazard regression was performed for baseline characteristics, including the classic cardiovascular risk factors, and confirms that age, LDL-cholesterol, and eGFR are associated with an increased risk for MACE (Supplementary Table 3).

**Table 2:** Clinical, laboratory, and angiographic characteristics of patients who reached the composite endpoint at 18 months follow-up.

**Parameter**	**Mace (*n* = 36)**	**Event-free group (*n* = 193)**	***p*-value**
**Clinical data**
Age (years)	67.8 ± 9.3	64.3 ± 8.4	0.02
Males, *n* (%)	25 (69.4)	132 (68.3)	0.90
BMI (kg/m^2^)	30.2 ± 3.7	29.1 ± 3.7	0.04
Heart rate (beats/min), median (Q1–Q3)	66.5 (60–70)	68 (60–75)	0.56
Arterial hypertension, *n* (%)	33 (91.6)	173 (89.6)	0.71
Diabetes, *n* (%)	16 (44.4)	65 (33.6)	0.21
**Laboratory data**
Glycemia (mg/dl), median (Q1–Q3)	106.1 (97–123.0)	108.3 (97.0–135.2)	0.77
Total cholesterol (mg/dl)	181.1 ± 54.8	165.5 ± 45.6	0.07
LDL-c (mg/dl)	102.7 ± 51.7	89.7 ± 33.4	0.08
HDL-c (mg/dl)	40.3 ± 11.8	43.3 ± 13.9	0.26
Creatinine (mg/dl), median (Q1–Q3)	1.1 (0.8–1.2)	0.9 (0.8–1.1)	0.02
eGFR (ml/min/1.73m^2^), median (Q1–Q3)	73.6 (59.9–87.4)	82.9 (70.8–101.9)	0.01
**Complete blood count**
Hemoglobin (g/dl)	13.3 ± 1.5	13.7 ± 1.4	0.10
Hematocrit (%)	39.3 ± 3.9	40.7 ± 3.8	0.05
Platelets/mm3	222,086 ± 49,414	242,963 ± 75,925	0.11
WBC/mm3	7,846 ± 2,084	7,628 ± 1,980	0.55
Neutrophils (%)	62.2 ± 9.9	61.1 ± 9.1	0.54
Lymphocytes (%)	26.4 ± 9.3	27.7 ± 7.7	0.36
Monocytes (%)	8.4 ± 3.8	8.1 ± 2.1	0.52
Basophils (%), median (Q1–Q3)	0.6 (0.4–0.7)	0.6 (0.4–0.8)	0.92
Eosinophils (%), median (Q1–Q3)	1.7 (0.9–3.0)	1.9 (1.1–3.0)	0.86
**Echocardiographic data**
LVEF (%), m ± SD	50.7 ± 10.1	53.4 ± 8.9	0.10
**Atherosclerosis severity**
SYNTAX Score I, median (Q1–Q3)	15.0 (7.7–24.5)	10.0 (7–19.2)	0.04
SYNTAX Score II – PCI, median (Q1–Q3)	33.7 (27.0–42.2)	28.4 (21.7–34.6)	0.006
SYNTAX Score II – CABG, median (Q1–Q3)	28.9 (20.1–37.6)	26.5 (20.0–33.1)	0.13

*Values are presented as mean ± SD unless otherwise stated*.

*BMI, body mass index; CABG, coronary artery bypass graft; eGFR, estimated glomerular filtration rate; HDL-c, high density lipoprotein cholesterol; LDL-c, low density lipoprotein cholesterol; LVEF, left ventricular ejection fraction; n, number; PCI, percutaneous coronary intervention; Q1, 1st quartile; Q3, 3rd quartile; WBC, white blood cells*.

### The Association Between Proteome and Major Adverse Cardiovascular Events

**Figure 1:**
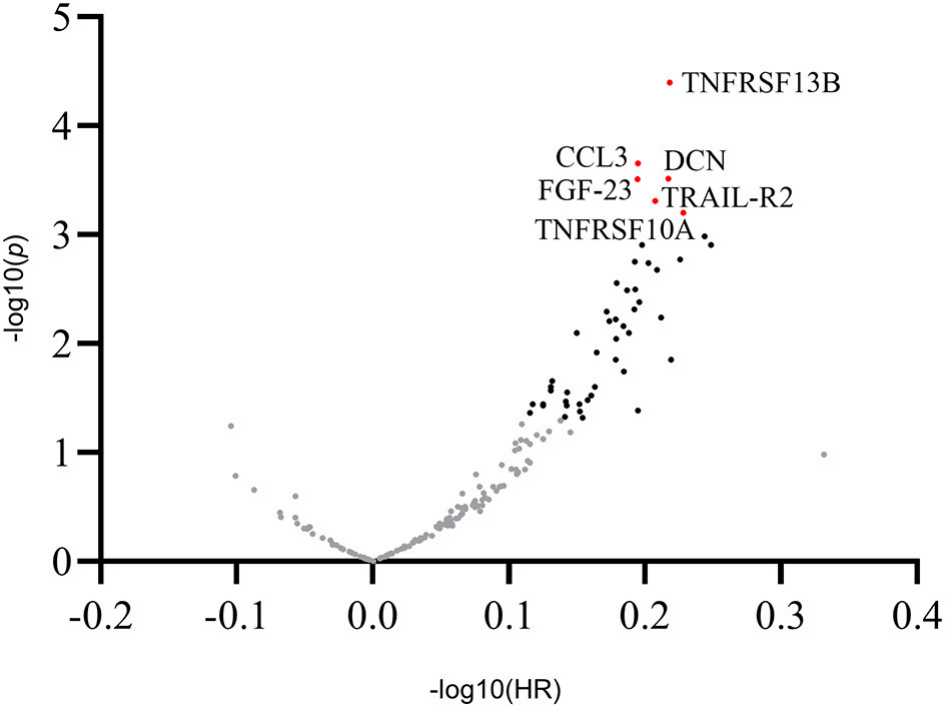
Overview of the hazard ratios of all proteins. Volcano plot showing log hazard ratios and *p*-values of plasma proteins for major adverse cardiovascular events in patients with stable coronary artery disease. Proteins associated with adverse outcomes at a level of significance of 0.05 are presented as black dots. Proteins with a statistically significant association with outcomes after adjusting for multiple comparisons (*p* < 0.001) are presented as labeled red dots. CCL3, C-C motif chemokine 3; DCN, decorin; FGF-23, fibroblast growth factor 23; TNFRSF13B, tumor necrosis factor receptor superfamily member 13B; TNFRSF10A, tumor necrosis factor receptor superfamily member 10A; TRAIL-R2, tumor necrosis factor-related apoptosis-inducing ligand receptor 2.

In univariate Cox proportional hazards regression analysis, of the selected 176 inflammatory biomarkers, 48 proteins were significantly associated with MACE (Supplementary Table 4). After adjusting for multiple comparisons, six proteins met the predefined significance criteria: tumor necrosis factor receptor superfamily member 13B (TNFRSF 13B) (HR = 1.65; 95% CI: 1.30–2.10; *p* < 0.001), C-C motif chemokine 3 (CCL3) (HR = 1.57; 95% CI: 1.23–1.99; *p* < 0.001), decorin (DCN) (HR = 1.65; 95% CI: 1.26–2.16; *p* < 0.001), fibroblast growth factor 23 (FGF-23) (HR = 1.56; 95% CI: 1.23–1.99; *p* < 0.001), tumor necrosis factor-related apoptosis-inducing ligand receptor 2 (TRAIL-R2) (HR = 1.61; 95% CI: 1.23–2.11; *p* < 0.001), and tumor necrosis factor receptor superfamily member 10A (TNFRSF10A) (HR = 1.69; 95% CI: 1.25–2.29; *p* < 0.001; Figure 1).

In *post-hoc* analysis, the following statistical power was obtained for each of the six mediators: 78.8% for TNFRSF13B and DCN, respectively, 82.3% for TNFRSF10A, 74.6% for TRAIL-R2, 70.0% for CCL3, and 68.7% for FGF-23, as individual parameters associated with MACE.

Multivariate Cox proportional-hazards regression analysis showed that only TNFRSF13B (HR = 1.53; 95% CI: 1.16–2.01; *p* = 0.002), CCL3 (HR = 1.40; 95% CI: 1.07–1.85; *p* = 0.01), DCN (HR = 1.43; 95% CI: 1.05–1.96; *p* = 0.02), FGF-23 (HR = 1.45; 95% CI: 1.08–1.93; *p* = 0.01), and TNFRSF10A (HR = 1.52; 95% CI: 1.05–2.18; *p* = 0.02) were associated with MACE independent of age, sex, diabetes mellitus, or eGFR (Table 3). Additional adjustment for LDL-cholesterol and arterial hypertension did not significantly change the point estimates.

**Table 3:** The relation between TNFRSF10A, TNFRSF13B, DCN, TRAIL-R2, CCL3, and FGF-23 and the risk of MACE.

**Biomarker**	**Model**					
	**I**		**II**		**III**	
	**HR (95% CI)**	***p*-value**	**HR (95% CI)**	***p*-value**	**HR (95% CI)**	***p*-value**
TNFRSF10A	1.69 (1.25–2.29)	<0.001	1.62 (1.18–2.22)	0.003	1.52 (1.05–2.18)	0.024
TNFRSF13B	1.65 (1.30–2.10)	<0.001	1.58 (1.23–2.04)	<0.001	1.53 (1.16–2.01)	0.002
DCN	1.65 (1.26–2.16)	<0.001	1.53 (1.13–2.06)	0.005	1.43 (1.05–1.96)	0.023
TRAIL-R2	1.61 (1.23–2.11)	<0.001	1.54 (1.15–2.05)	0.003	1.44 (0.99–2.11)	0.056
CCL3	1.57 (1.23–1.98)	<0.001	1.48 (1.15–1.91)	0.002	1.40 (1.07–1.85)	0.015
FGF-23	1.56 (1.23–1.99)	<0.001	1.53 (1.19–1.97)	<0.001	1.45 (1.08–1.93)	0.012

*Hazard ratios represent risk per 1 SD increase in each biomarker*.

*Model I = Univariable model*.

*Model II = Model I adjusted for age and sex*.

*Model III = Model II adjusted for diabetes mellitus and eGFR*.

*CCL3, C-C motif chemokine 3; DCN, decorin; eGFR, estimated glomerular filtration rate; FGF-23, Fibroblast growth factor 23; MACE, major adverse cardiovascular events; TNFRSF10A, tumor necrosis factor receptor superfamily member 10A; TNFRSF13B, tumor necrosis factor receptor superfamily member 13B; TRAIL-R2, TNF-related apoptosis-inducing ligand receptor 2*.

### Relation Between Proteome and Cardiovascular Risk Factors

**Figure 2:**
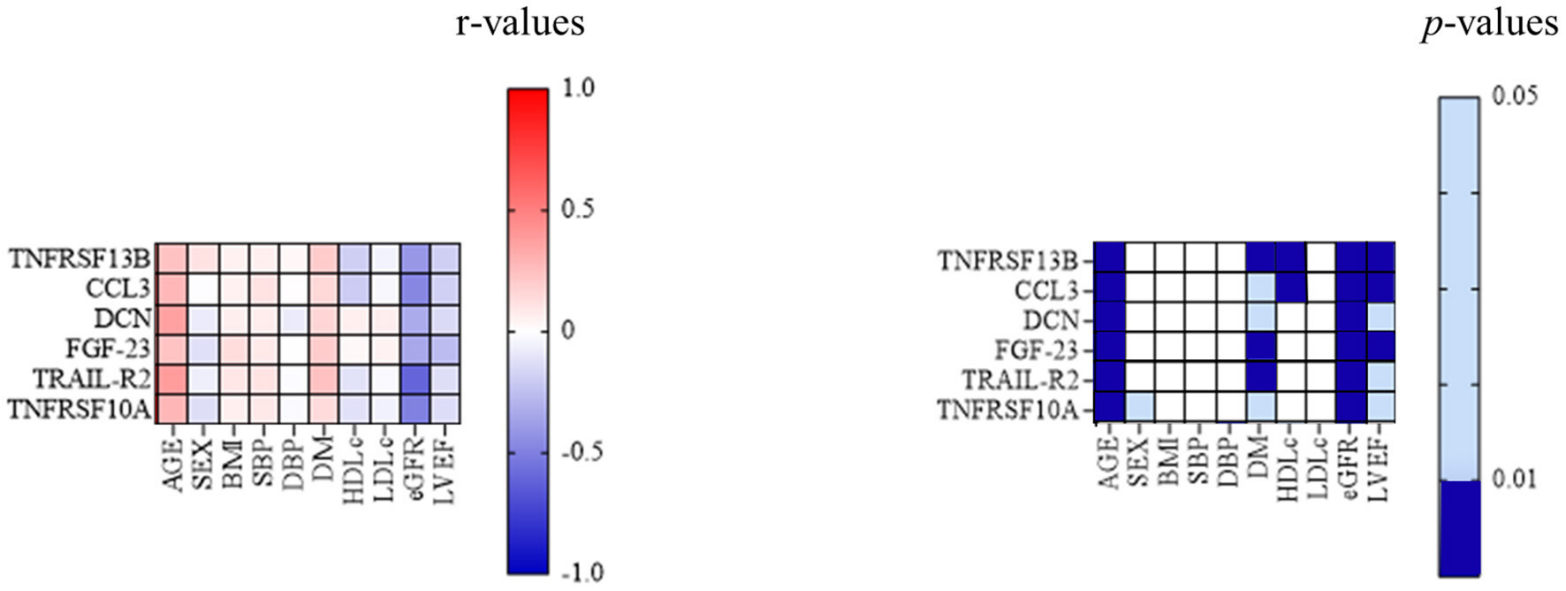
Associations between classic cardiovascular risk factors and inflammatory biomarkers. The correlation between TNFRSF13B, CCL3, DCN, FGF-23, TRAIL-R2, and TNFRSF10A, and the classic cardiovascular risk factors; *p*-value 0.01–0.05—light blue; *p* < 0.01—dark blue. BMI, body mass index; CCL3, C-C motif chemokine 3; DCN, decorin; DBP, diastolic blood pressure; DM, diabetes mellitus; eGFR, estimated glomerular filtration rate; FGF-23, fibroblast growth factor 23; HDLc, high-density lipoprotein cholesterol; LDLc, low-density lipoprotein cholesterol; LVEF, left ventricular ejection fraction; MACE, major adverse cardiovascular events; SBP, systolic blood pressure; TNFRSF10A, tumor necrosis factor receptor superfamily member 10A; TNFRSF 13B, tumor necrosis factor receptor superfamily member 13B; TRAIL-R2, tumor necrosis factor-related apoptosis-inducing ligand receptor 2.

When studying the association between TNFRSF 13B, CCL3, DCN, FGF-23, TRAIL-R2, and TNFRSF10A and classic cardiovascular risk factors, all six markers correlated positively with age (*p* < 0.001 for each association) and diabetes mellitus (*p* = 0.002 for TNFRSF13B, 0.02 for CCL3, 0.01 for DCN, 0.002 for FGF-23, <0.001 for TRAIL-R2, and 0.03 for TNFRSF10A), while only TNFRSF 13B and CCL3 correlated negatively with HDL-cholesterol (*p* = 0.008 and 0.004, respectively). All six proteins correlated negatively with both eGFR (*p* < 0.001 for each association) and left ventricular ejection fraction (LVEF) (*p* = 0.004 for TNFRSF13B and CCL3, 0.02 for DCN, 0.001 for FGF-23, 0.04 for TRAIL-R2, and 0.03 for TNFRSF10A) (Figure 2).

### The Association Between Proteome and Coronary Atherosclerosis

Positive correlations were identified between the extent and complexity of atherosclerosis at the moment of inclusion, as defined by SYNTAX I Score, and IL-18 (*r* = 0.15, *p* = 0.04), fatty-acid binding protein 2, intestinal (FABP2) (*r* = 0.18, *p* = 0.01), gastrotropin (GT) (*r* = 0.21, *p* = 0.004), natriuretic peptides B (BNP) (*r* = 0.21, *p* = 0.006), cathepsin L1 (CTSL1) (*r* = 0.16, *p* = 0.03), and C-C motif chemokine 25 (CCL25) (*r* = 0.16, *p* = 0.03), respectively.

## Discussion

The present study aimed to find circulating proteins that are associated with the occurrence of a first MACE in patients with chronic stable CAD. Therefore, we simultaneously assessed a large panel of circulating proteins in a prospective cohort study of patients with stable CAD, with angiographically documented coronary atherosclerosis and inducible myocardial ischemia. We identified six inflammatory biomarkers from plasma that were significantly associated with a first MACE after adjusting for multiple comparisons: TNFRSF13B, CCL3, DCN, FGF-23, TRAIL-R2, and TNFRSF10A. Except for TRAIL-R2, the other five proteins were identified as predictors of MACE independent of age, sex, diabetes mellitus, or eGFR. The relevance of this finding is two-fold. First, these proteins help to understand the pathophysiology of atherothrombosis. Secondly, these proteins could act as biomarkers to improve individual risk prediction, which has to be tested in independent cohorts added to known risk prediction models.

One pathway through which inflammation can impact CVD evolution is through circulating inflammatory proteins. Although numerous plasma proteins have been studied in relation to plaque morphology, supporting data remains limited about the use of proteomics in identifying patients with stable CAD at risk of developing future MACE.

Of the five proteins independently associated with MACE, TNFRSF13B, and TNFRSF10A are involved in the TNF signaling pathway, and have roles in apoptosis, NFkB activation, and B-cell activation (17, 18).

Recently, in a cross-sectional study in patients with suspected CAD, TNFRSF13B/TACI (transmembrane activator and CAML interactor) was identified as predictive for the presence of high-risk atherosclerotic plaques on coronary computed tomographic angiography (10). TNFRSF13B is a member of the TNF receptor superfamily and one of the three receptors for BAFF (B cell activating factor of the TNF family). In addition to BAFF, TNFRSF13B is also bound by the alternative ligand APRIL (A Proliferation Inducing Ligand), a closely related BAFF homolog (19). Importantly, membrane-bound TNFRSF13B/TACI can be cleaved by ADAM10 (A Disintegrin and metalloproteinase domain-containing protein 10), and soluble TACI (sTACI) has been associated with B-cell activation (20). sTACI can act as a decoy receptor for circulating BAFF.

Given the multiple roles of TNFRSF13B in experimental models of atherogenesis, it is not surprising that reports on the association between TNFRSF13B and CVD are conflicting. It was demonstrated that high circulating concentrations of BAFF in patients with acute myocardial infarction predicted an increased risk of death or recurrent myocardial infarction (21). On the contrary, recent data raised concern about the potential cardiovascular hazard that could accompany BAFF blockade in chronic cardiovascular inflammatory settings, in contrast to acute cardiovascular inflammation (22). The complexity of the BAFF-APRIL system and the precise effects of anti-BAFF immunotherapy on atherosclerosis remain yet to be fully described. We hypothesize that the previous association between circulating TNFRSF13B and high-risk coronary atherosclerotic plaques, and the predictive effect of TNFRSF13B for MACE development in our study, are due to decoy function of soluble TNFRSF13B of binding circulating BAFF which limits its atheroprotective effects in plaques. This potential mechanism should be further explored in future studies.

Another member of the TNF-receptor superfamily identified as predictor of MACE was TNFRSF10A. TNFRSF10A modulates apoptosis and proliferation of vascular smooth muscle cells (17, 23). Depending on the stage of an atherosclerotic plaque, these processes could have both beneficial and detrimental effects on lesion progression (24). A previous study has identified TNFRSF10A as predictor of recurrent acute events in patients with a history of acute coronary syndromes (24), while in another study TNFRSF10A was associated with the presence of high-risk coronary atherosclerotic plaques on computed tomography angiography (10). Although, the debate is currently ongoing regarding the importance of TNFRSF10A as a predictor for atherosclerosis progression, our results come to support the accumulated past evidence.

Regarding the association between TNFRSF13B, TNFRSF10A, and type II diabetes mellitus, our results support previous findings of TNF-α being linked to obesity and insulin resistance (25).

Another biomarker associated with adverse outcomes in our study was CCL3. CCL3, also known as macrophage inflammatory protein-1α, belongs to the CC chemokine family and is involved in leukocyte recruitment and activation, and has been identified in human atherosclerotic lesions (26). CCL3 is mainly released by activated macrophages, but also by activated platelets, neutrophils, and mast cells, contributing to chemotaxis in atherothrombosis (27). CCL3 attracts different leukocyte subsets, accelerates lesion formation, and is an independent predictor of future myocardial ischemia (28). In one previous study in patients with acute coronary syndromes, CCL3 was associated with an increased risk of fatal events during follow-up (29).

Existing literature data is more conflicting regarding decorin, a small leucine-rich proteoglycan, which induces calcification of arterial smooth muscle cells and localizes to mineral deposits in human atherosclerotic plaques (30). Decorin is supposed to function as a promoter of intimal calcification and was also associated with calcium deposits in lesions from sudden death patients (31). It was suggested that macrophages may cause increased calcification of atherosclerotic plaques by inducing decorin expression through IL-1 release (31). In our study, high decorin levels were independently associated with MACE development, an observation consistent with the previous finding that decorin is associated with advanced atherosclerotic plaques (32). On the contrary, other authors concluded that systemic overexpression of decorin reduced inflammation and fibrosis in the atherosclerotic plaques of atherosclerosis-prone mice and slowed disease progression (33). In fact, in another study, staining for decorin was weak or non-existent at the site of atherosclerotic plaque rupture (34). Although decorin can promote the formation of highly ordered structures of fibrillar proteins, it may also play a role in destroying these fibrillar systems, through the stimulation of collagenase expression (35). The controversial roles decorin has in atherosclerotic disease progression remain to be further clarified. Because decorin has a glucose response element in its promoter, it could play a role in severe vascular calcifications occurring in diabetic patients (31). In fact, in our study, decorin was also associated with the presence of diabetes mellitus.

The last biomarker independently associated with MACE in our cohort was FGF-23, a phosphorus regulating protein secreted by osteoblasts (36) and a recently described risk factor for cardiovascular disease (37). The association we identified between FGF-23 and atherosclerotic MACE comes to support these previous findings. A possible causal relationship was also described between FGF-23 and adverse cardiovascular remodeling, involving left ventricular hypertrophy, renin-angiotensin system upregulation and the promotion of vascular calcification (38). In a large cohort of patients with recent acute coronary syndromes, an elevated level of FGF-23 was associated with the risk of adverse outcomes, including cardiovascular death or heart failure hospitalization (38). These findings supported the results of another study in post-acute coronary syndrome patients, in which elevated FGF-23 concentrations were associated with a higher incidence of the composite outcome, including ischemic events, heart failure, and all-cause mortality (39).

Regarding the association between plasma proteins and the extent and complexity of coronary atherosclerosis, a positive correlation was identified between SYNTAX I Score and various proteins. Interestingly, different proteins showed associations with atherosclerosis severity vs. the occurrence of MACE, suggesting that these are different pathophysiological processes.

Despite recent advances in the control of classic cardiovascular risk factors, patients with CAD remain at increased risk of acute adverse events. Several inflammatory biomarkers, such as hs-CRP, have been proposed to quantify the extent of residual risk and to guide patient-tailored therapeutic strategies (3), but current guidelines do not recommend their routine use in clinical practice (40). Extensive literature data exists on the association between inflammation and cardiovascular disease. However, aggressive lipid-lowering therapy remains the main secondary prevention strategy in patients with documented CAD. Still, in some patients, even aggressive LDL-cholesterol lowering with intensive statin therapy, ezetimibe, or monoclonal antibodies inhibiting proprotein convertase subtilisin-kexin type 9 is of limited benefit on the recurrence of acute cardiovascular events, given the inflammation-driven proportion of residual risk (41). Hs-CPR can be used as a marker for systemic inflammation, but it is non-specific, and is not directly involved in the pathogenesis of atherosclerotic plaque destabilization. The findings of our study could help to develop novel specific anti-inflammatory treatments and to improve measures of residual inflammatory risk. Therefore, future studies should investigate the causal roles of the identified proteins in atherothrombosis in experimental models, and address the predictive value of the proteins on top of existing risk prediction models in independent cohorts.

Our study selected a well-defined cohort of patients with stable CAD, in which guideline-based secondary prevention measures were implemented. Two-thirds of the included patients had a history of elective coronary revascularization and more than 90% of the patients were on statin therapy. We cannot exclude that statin treatment influenced the expression of the five proteomic biomarkers we identified to be associated with the development of a first-ever MACE. However, the proportion of patients receiving statins did not differ in the MACE group as compared to the event-free population, therefore, their potential effect on the expression of the proteomic biomarkers was consistent throughout the cohort. In this setting, our study brings evidence on the potential role of five novel circulating proteins as risk predictors and possible pharmacological targets in patients with stable CAD.

Limitations of the study need to be considered and include the relatively small sample size. Therefore, these results should be seen as hypothesis-generating. Validation in larger prospective, secondary prevention cohorts is necessary to establish their value for clinical application. Another limitation of our study consists in the use of a restrictive 2.5% FDR, which increases the probability of false-negative results. However, because of the large number of biomarkers to be tested, our objective was to reduce the probability of a type I error.

In conclusion, five proteomic biomarkers, including two related to the TNF pathway, were identified in a cohort of patients with stable CAD, as independent risk factors for the occurrence of a first MACE: TNFRSF13B, CCL3, DCN, FGF-23, and TNFRSF10A. In addition to guideline-based secondary prevention measures, novel biomarkers could represent pharmacological targets for the prevention of adverse cardiovascular events.

## Data Availability Statement

The datasets presented in this study can be found in online repositories. The names of the repository/repositories and accession number(s) can be found below: PeptideAtlas, accession no: PASS01721.

## Ethics Statement

The studies involving human participants were reviewed and approved by the Ethics Committee of Iuliu Haţieganu University of Medicine and Pharmacy, Cluj-Napoca, Romania. The patients/participants provided their written informed consent to participate in this study.

## Author Contributions

MD, AŢ, LJ, MN, NR, and AI contributed to the conception, design of the work, acquisition, analysis, and interpretation of data. SB, FV, SB, and CH contributed to the analysis and interpretation of data. All authors drafted the work, revised it critically for important intellectual content, gave final approval of the version to be published, and agreed to be accountable for all aspects of the work.

## Funding

HORIZON 2020 European Research Program—REPROGRAM: Targeting epigenetic REPROGRamming of innate immune cells in Atherosclerosis Management and other chronic inflammatory diseases (grant agreement no. 667837). MN was supported by an ERC Advanced Grant (#833247) and a Spinoza grant of the Netherlands Organization for Scientific Research. NR is recipient of a grant of the ERA-CVD Joint Transnational Call 2018, which is supported by the Dutch Heart Foundation (JTC2018, project MEMORY; 2018T093). NR, LJ, and MN received an IN-CONTROL CVON grant from the Dutch Heart Foundation (CVON2012-03 and CVON2018-27). LJ is supported by a Competitiveness Operational Programme grant of the Romanian Ministry of European Funds [HINT, ID P_37_762; MySMIS 103587]. SB is funded by the Dutch Heart Foundation (Dekker grant, grant no. 2018T028). The sources of funding had no role in the design and conduct of the study, in the collection, management, analysis, and interpretation of the data, in the preparation, review, or approval of the manuscript, and in the decision to submit the manuscript for publication.

## Conflict of Interest

The authors declare that the research was conducted in the absence of any commercial or financial relationships that could be construed as a potential conflict of interest.

## Publisher's Note

All claims expressed in this article are solely those of the authors and do not necessarily represent those of their affiliated organizations, or those of the publisher, the editors and the reviewers. Any product that may be evaluated in this article, or claim that may be made by its manufacturer, is not guaranteed or endorsed by the publisher.

## References

[1] Libby P Ridker PM Maseri A. Inflammation and atherosclerosis. Circulation. (2002) 105:1135–43. 10.1161/hc0902.104353 11877368

[2] Ridker PM Hennekens CH Buring JE Rifai N. C-reactive protein and other markers of inflammation in the prediction of cardiovascular disease in women. N Engl J Med. (2000) 342:836–43. 10.1056/NEJM200003233421202 10733371

[3] Ridker PM Everett BM Thuren T MacFadyen JG Chang WH Ballantyne C. Antiinflammatory therapy with canakinumab for atherosclerotic disease. N Engl J Med. (2017) 377:1119–31. 10.1056/NEJMoa1707914 28845751

[4] Tardif JC Kouz S Waters DD Bertrand OF Diaz R Maggioni AP. Efficacy and safety of low-dose colchicine after myocardial infarction. N Engl J Med. (2019) 381:2497–505. 10.1056/NEJMoa1912388 31733140

[5] Nidorf SM Fiolet ATL Mosterd A Eikelboom JW Schut A Opstal TSJ. Colchicine in patients with chronic coronary disease. N Engl J Med. (2020) 383:1838–47. 10.1056/NEJMoa2021372 32865380

[6] Eichler K Puhan MA Steurer J Bachmann LM. Prediction of first coronary events with the Framingham score: a systematic review. Am Heart J. (2007) 153:722–31. 10.1016/j.ahj.2007.02.027 17452145

[7] Lindholm D Lindbäck J Armstrong PW Budaj A Cannon CP Granger CB. Biomarker-based risk model to predict cardiovascular mortality in patients with stable coronary disease. J Am Coll Cardiol. (2017) 70:813–26. 10.1016/j.jacc.2017.06.030 28797349

[8] Oemrawsingh RM Cheng JM García-García HM Kardys I van Schaik RH Regar E. High-sensitivity Troponin T in relation to coronary plaque characteristics in patients with stable coronary artery disease; results of the ATHEROREMO-IVUS study. Atherosclerosis. (2016) 247:135–41. 10.1016/j.atherosclerosis.2016.02.012 26917225

[9] Caselli C De Graaf MA Lorenzoni V Rovai D Marinelli M Del Ry S. HDL cholesterol, leptin and interleukin-6 predict high risk coronary anatomy assessed by CT angiography in patients with stable chest pain. Atherosclerosis. (2015) 241:55–61. 10.1016/j.atherosclerosis.2015.04.811 25966440

[10] Bom MJ Levin E Driessen RS Danad I Van Kuijk CC van Rossum AC. Predictive value of targeted proteomics for coronary plaque morphology in patients with suspected coronary artery disease. EBioMedicine. (2019) 39:109–17. 10.1016/j.ebiom.2018.12.033 30587458 PMC6355456

[11] Hoogeveen RM Pereira JPB Nurmohamed NS Zampoleri V Bom MJ Baragetti A. Improved cardiovascular risk prediction using targeted plasma proteomic in primary prevention. Eur Heart J. (2020) 41:3998–4007. 10.1093/eurheartj/ehaa648 32808014 PMC7672529

[12] Williams B Mancia G Spiering W Agabiti Rosei E Azizi M Burnier M. 2018 ESC/ESH Guidelines for the management of arterial hypertension. Eur Heart J. (2018) 39:3021–104. 10.1093/eurheartj/ehy339 30165516

[13] World Health Organization. Definition and Diagnosis of Diabetes Mellitus and Intermediate and Hyperglycaemia. Report of a WHO/IDF Consultation. Available online at: http://www.who.int/diabetes/publications/diagnosis_diabetes2006/en (accessed October 10, 2020).

[14] World Health Organization. Use of Glycated Haemoglobin (HbA1c) in the Diagnosis of Diabetes Mellitus: Abbreviated Report of a WHO Consultation. Available online at: http://www.who.int/diabetes/publications/report-hba1c_2011.pdf (accessed October 10, 2020). 26158184

[15] Assarsson E Lundberg M Holmquist G Björkesten J Thorsen S Ekman D. Homogenous 96-plex PEA immunoassay exhibiting high sensitivity, specificity, and excellent scalability. PLoS One. (2014) 9:e95192. 10.1371/journal.pone.0095192 24755770 PMC3995906

[16] Benjamini Y Hochberg Y. Controlling the false discovery rate: a practical and powerful approach to multiple testing. J R Stat Soc. (1995) 57:289–300. 10.1111/j.2517-6161.1995.tb02031.x

[17] Kavurma MM Tan NY Bennett MR. Death receptors and their ligands in atherosclerosis. Arterioscler Thromb Vasc Biol. (2008) 28:1694–702. 10.1161/ATVBAHA.107.155143 18669890

[18] Cheng W Zhao Y Wang S Jiang F. Tumor necrosis factor-related apoptosis-inducing ligand in vascular inflammation and atherosclerosis: a protector or culprit? Vascul Pharmacol. (2014) 63:135–44. 10.1016/j.vph.2014.10.004 25451562

[19] Mackay F Schneider P. Cracking the BAFF code. Nat Rev Immunol. (2009) 9:491–502. 10.1038/nri2572 19521398

[20] Hoffmann FS Kuhn PH Laurent SA Hauck SM Berer K Wendlinger SA. The immunoregulator soluble TACI is released by ADAM10 and reflects B cell activation in autoimmunity. J Immunol. (2015) 194:542–52. 10.4049/jimmunol.1402070 25505277 PMC4282951

[21] Zouggari Y Ait-Oufella H Bonnin P Simon T Sage AP Guérin C. B lymphocytes trigger monocyte mobilization and impair heart function after acute myocardial infarction. Nat Med. (2013) 19:1273–80. 10.1038/nm.3284 24037091 PMC4042928

[22] Tsiantoulas D Sage AP Göderle L Ozsvar-Kozma M Murphy D Porsch F. B cell-activating factor neutralization aggravates atherosclerosis. Circulation. (2018) 138:2263–73. 10.1161/CIRCULATIONAHA.117.032790 29858401 PMC6181204

[23] Kavurma MM Bennett MR. Expression, regulation and function of trail in atherosclerosis. Biochem Pharmacol. (2008) 75:1441–50. 10.1016/j.bcp.2007.10.020 18061141

[24] Vroegindewey MM van den Berg VJ Oemrawsingh RM Kardys I Asselbergs FW van der Harst P. The temporal pattern of immune and inflammatory proteins prior to a recurrent coronary event in post-acute coronary syndrome patients. Biomarkers. (2019) 24:199–205. 10.1080/1354750X.2018.1539768 30514120

[25] Hotamisligil GS Shargill NS Spiegelman BM. Adipose expression of tumor necrosis factor-alpha: direct role in obesity-linked insulin resistance. Science. (1993) 259:87–91. 10.1126/science.7678183 7678183

[26] Reape TJ Rayner K Manning CD Gee AN Barnette MS Burnand KG. Expression and cellular localization of the CC chemokines PARC and ELC in human atherosclerotic plaques. Am J Pathol. (1999) 154:365–74. 10.1016/S0002-9440(10)65283-2 10027395 PMC1850009

[27] Weber C. Platelets and chemokines in atherosclerosis: partners in crime. Circ Res. (2005) 96:612–6. 10.1161/01.RES.0000160077.17427.57 15802619

[28] de Jager SC Bot I Kraaijeveld AO Korporaal SJ Bot M van Santbrink PJ. Leukocyte-specific CCL3 deficiency inhibits atherosclerotic lesion development by affecting neutrophil accumulation. Arterioscler Thromb Vasc Biol. (2013) 33:e75–e83. 10.1161/ATVBAHA.112.300857 23288165

[29] de Jager SC Bongaerts BW Weber M Kraaijeveld AO Rousch M Dimmeler S. Chemokines CCL3/MIP1α, CCL5/RANTES and CCL18/PARC are independent risk predictors of short-term mortality in patients with acute coronary syndromes. PLoS ONE. (2012) 7:e45804. 10.1371/journal.pone.0045804 23029252 PMC3448678

[30] Nazemi S Rezapour A Moallem SMH Afshar M Elyasi S Mashreghi Moghadam HR. Could decorin be a biomarker of coronary artery disease? A pilot study in human beings. Acta Biomed. (2018) 89:365–9. 10.23750/abm.v89i3.6024 30333460 PMC6502117

[31] Fischer JW Steitz SA Johnson PY Burke A Kolodgie F Virmani R. Decorin promotes aortic smooth muscle cell calcification and colocalizes to calcified regions in human atherosclerotic lesions. Arterioscler Thromb Vasc Biol. (2004) 24:2391–6. 10.1161/01.ATV.0000147029.63303.28 15472131

[32] Gutierrez P O'Brien KD Ferguson M Nikkari ST Alpers CE Wight TN. Differences in the distribution of versican, decorin, and biglycan in atherosclerotic human coronary arteries. Cardiovasc Pathol. (1997) 6:271–8. 10.1016/S1054-8807(97)00001-X 25989722

[33] Al Haj Zen A Caligiuri G Sainz J Lemitre M Demerens C Lafont A. Decorin overexpression reduces atherosclerosis development in apolipoprotein E-deficient mice. Atherosclerosis. (2006) 187:31–9. 10.1016/j.atherosclerosis.2005.08.023 16183063

[34] Kolodgie FD Burke AP Farb A Weber DK Kutys R Wight TN. Differential accumulation of proteoglycans and hyaluronan in culprit lesions: insights into plaque erosion. Arterioscler Thromb Vasc Biol. (2002) 22:1642–8. 10.1161/01.ATV.0000034021.92658.4C 12377743

[35] Järveläinen H Sainio A Wight TN. Pivotal role for decorin in angiogenesis. Matrix Biol. (2015) 43:15–26. 10.1016/j.matbio.2015.01.023 25661523 PMC4560244

[36] Seiler S Heine GH Fliser D. Clinical relevance of FGF-23 in chronic kidney disease. Kidney Int Suppl. (2009) 114:S34–S42. 10.1038/ki.2009.405 19946326

[37] Scialla JJ Xie H Rahman M Anderson AH Isakova T Ojo A. Fibroblast growth factor-23 and cardiovascular events in CKD. J Am Soc Nephrol. (2014) 25:349–60. 10.1681/ASN.2013050465 24158986 PMC3904568

[38] Bergmark BA Udell JA Morrow DA Cannon CP Steen DL Jarolim P. Association of fibroblast growth factor 23 with recurrent cardiovascular events in patients after an acute coronary syndrome: a secondary analysis of a randomized clinical trial. JAMA Cardiol. (2018) 3:473–80. 10.1001/jamacardio.2018.0653 29710336 PMC6128514

[39] Tuñón J Cristóbal C Tarín N Aceña Á González-Casaus ML Huelmos A. Coexistence of low vitamin D and high fibroblast growth factor-23 plasma levels predicts an adverse outcome in patients with coronary artery disease. PLoS One. (2014) 9:e95402. 10.1371/journal.pone.0095402 24748388 PMC3991663

[40] Piepoli MF Hoes AW Agewall S Albus C Brotons C Catapano AL. 2016 European Guidelines on cardiovascular disease prevention in clinical practice: The Sixth Joint Task Force of the European Society of Cardiology and Other Societies on Cardiovascular Disease Prevention in Clinical Practice (constituted by representatives of 10 societies and by invited experts). Developed with the special contribution of the European Association for Cardiovascular Prevention & Rehabilitation (EACPR). Eur Heart J. (2016). 37:2315–81. 10.1093/eurheartj/ehw106 27222591 PMC4986030

[41] Aday AW Ridker PM. Targeting residual inflammatory risk: a shifting paradigm for atherosclerotic disease. Front Cardiovasc Med. (2019) 6:16. 10.3389/fcvm.2019.00016 30873416 PMC6403155

