# Supplementary material for: Relation Between Plasma Proteomics Analysis and Major Adverse Cardiovascular Events in Patients With Stable Coronary Artery Disease
Source: Front Cardiovasc Med. 2022 Feb 8;9:731325. doi: 10.3389/fcvm.2022.731325 (PMC8861429; doi:10.3389/fcvm.2022.731325)
Supplement: Supplementary file 1 [file Data_Sheet_1.docx]

Supplementary Material

# Supplementary Tables

# Table S1. List of all measured proteins (n=177)

| No | Protein | Abbreviation | UniProt ID |
| --- | --- | --- | --- |
| 1 | 2,4-dienoyl-CoA reductase, mitochondrial | DECR1 | Q16698 |
| 2 | A disintegrin and metalloproteinase with thrombospondin motifs 13 | ADAM-TS13 | Q76LX8 |
| 3 | Adenosine Deaminase | ADA | P00813 |
| 4 | ADM | ADM | P35318 |
| 5 | Agouti-related protein (AGRP) | AGRP | O00253 |
| 6 | Alpha-L-iduronidase (IDUA) | IDUA | P35475 |
| 7 | Angiopoietin-1 (ANGPT1) | ANG-1 | Q15389 |
| 8 | Angiopoietin-1 receptor (TIE2) | TIE2 | Q02763 |
| 9 | Angiotensin-converting enzyme 2 (ACE2) | ACE2 | Q9BYF1 |
| 10 | Artemin | ARTN | Q5T4W7 |
| 11 | Axin-1 | AXIN1 | O15169 |
| 12 | Beta-nerve growth factor | Beta-NGF | P01138 |
| 13 | Bone morphogenetic protein 6 (BMP-6) | BMP-6 | P22004 |
| 14 | Brother of CDO (BOC) | BOC | Q9BWV1 |
| 15 | Carbonic anhydrase 5A, mitochondrial (CA5A) | CA5A | P35218 |
| 16 | Carcinoembryonic antigenrelated cell adhesion molecule 8 (CEACAM8) | CEACAM8 | P31997 |
| 17 | Caspase-8 | CASP-8 | Q14790 |
| 18 | Cathepsin L1 (CTSL1) | CTSL1 | P07711 |
| 19 | C-C motif chemokine 17 (CCL17) | CCL17 | Q92583 |
| 20 | C-C motif chemokine 19 | CCL19 | Q99731 |
| 21 | C-C motif chemokine 20 | CCL20 | P78556 |
| 22 | C-C motif chemokine 23 | CCL23 | P55773 |
| 23 | C-C motif chemokine 25 | CCL25 | O15444 |
| 24 | C-C motif chemokine 28 | CCL28 | Q9NRJ3 |
| 25 | C-C motif chemokine 3 (CCL3) | CCL3 | P10147 |
| 26 | C-C motif chemokine 4 | CCL4 | P13236 |
| 27 | CD40 ligand (CD40-L) | CD40-L | P29965 |
| 28 | CD40L receptor | CD40 | P25942 |
| 29 | Chymotrypsin C (CTRC) | CTRC | Q99895 |
| 30 | CUB domain-containing protein 1 | CDCP1 | Q9H5V8 |
| 31 | C-X-C motif chemokine 1 (CXCL1) | CXCL1 | P09341 |
| 32 | C-X-C motif chemokine 10 | CXCL10 | P02778 |
| 33 | C-X-C motif chemokine 11 | CXCL11 | O14625 |
| 34 | C-X-C motif chemokine 5 | CXCL5 | P42830 |
| 35 | C-X-C motif chemokine 6 | CXCL6 | P80162 |
| 36 | C-X-C motif chemokine 9 | CXCL9 | Q07325 |
| 37 | Cystatin D | CST5 | P28325 |
| 38 | Decorin (DCN) | DCN | P07585 |
| 39 | Delta and Notch-like epidermal growth factor-related receptor | DNER | Q8NFT8 |
| 40 | Dickkopf-related protein 1 (Dkk-1) | Dkk-1 | O94907 |
| 41 | Eotaxin | CCL11 | P51671 |
| 42 | Eukaryotic translation initiation factor 4E-binding protein 1 | 4E-BP1 | Q13541 |
| 43 | Fatty acid-binding protein, intestinal (FABP2) | FABP2 | P12104 |
| 44 | Fibroblast growth factor 19 | FGF-19 | O95750 |
| 45 | Fibroblast growth factor 21 (FGF21) | FGF-21 | Q9NSA1 |
| 46 | Fibroblast growth factor 23 (FGF-23) | FGF-23 | Q9GZV9 |
| 47 | Fibroblast growth factor 5 | FGF-5 | Q8NF90 |
| 48 | Fms-related tyrosine kinase 3 ligand | Flt3L | P49771 |
| 49 | Follistatin (FS) | FS | P19883 |
| 50 | Fractalkine | CX3CL1 | P78423 |
| 51 | Galectin-9 (Gal-9) | Gal-9 | O00182 |
| 52 | Gastric intrinsic factor (GIF) | GIF | P27352 |
| 53 | Gastrotropin (GT) | GT | P51161 |
| 54 | Glial cell line-derived neurotrophic factor | GDNF | P39905 |
| 55 | Growth hormone (GH) | GH | P01241 |
| 56 | Growth/differentiation factor 2 (GDF-2) | GDF-2 | Q9UK05 |
| 57 | Heat shock 27 kDa protein | HSP 27 | P04792 |
| 58 | Heme oxygenase 1 | HO-1 | P09601 |
| 59 | Hepatocyte growth factor | HGF | P14210 |
| 60 | Hydroxyacid oxidase 1 | HAOX1 | Q9UJM8 |
| 61 | Interferon gamma | IFN-gamma | P01579 |
| 62 | Interleukin-1 alpha | IL-1 alpha | P01583 |
| 63 | Interleukin-1 receptor antagonist protein | IL-1ra | P18510 |
| 64 | Interleukin-1 receptor-like 2 | IL1RL2 | Q9HB29 |
| 65 | Interleukin-10 | IL10 | P22301 |
| 66 | Interleukin-10 receptor subunit alpha | IL-10RA | Q13651 |
| 67 | Interleukin-10 receptor subunit beta | IL-10RB | Q08334 |
| 68 | Interleukin-12 subunit beta | IL-12B | P29460 |
| 69 | Interleukin-13 | IL-13 | P35225 |
| 70 | Interleukin-15 receptor subunit alpha | IL-15RA | Q13261 |
| 71 | Interleukin-17A | IL-17A | Q16552 |
| 72 | Interleukin-17C | IL-17C | Q9P0M4 |
| 73 | Interleukin-17D | IL-17D | Q8TAD2 |
| 74 | Interleukin-18 | IL-18 | Q14116 |
| 75 | Interleukin-18 receptor 1 | IL-18R1 | Q13478 |
| 76 | Interleukin-2 | IL-2 | P60568 |
| 77 | Interleukin-2 receptor subunit beta | IL-2RB | P14784 |
| 78 | Interleukin-20 | IL-20 | Q9NYY1 |
| 79 | Interleukin-20 receptor subunit alpha | IL-20RA | Q9UHF4 |
| 80 | Interleukin-22 receptor subunit alpha-1 | IL-22 RA1 | Q8N6P7 |
| 81 | Interleukin-24 | IL-24 | Q13007 |
| 82 | Interleukin-27 | IL-27 | Q8NEV9; Q14213 |
| 83 | Interleukin-33 | IL-33 | O95760 |
| 84 | Interleukin-4 | IL-4 | P05112 |
| 85 | Interleukin-4 receptor subunit alpha | IL-4RA | P24394 |
| 86 | Interleukin-5 | IL-5 | P05113 |
| 87 | Interleukin-6 | IL-6 | P05231 |
| 88 | Interleukin-7 | IL-7 | P13232 |
| 89 | Interleukin-8 | IL-8 | P10145 |
| 90 | Kidney Injury Molecule | KIM1 | Q96D42 |
| 91 | Lactoylglutathione lyase | GLO1 | Q04760 |
| 92 | Latency-associated peptide transforming growth factor beta-1 | LAP TGF-beta-1 | P01137 |
| 93 | Lectin-like oxidized LDL receptor 1 | LOX-1 | P78380 |
| 94 | Leptin | LEP | P41159 |
| 95 | Leukemia inhibitory factor | LIF | P15018 |
| 96 | Leukemia inhibitory factor receptor | LIF-R | P42702 |
| 97 | Lipoprotein lipase | LPL | P06858 |
| 98 | Low affinity immunoglobulin gamma Fc region receptor II-b | IgG Fc receptor II-b | P31994 |
| 99 | Lymphotactin | XCL1 | P47992 |
| 100 | Macrophage colony-stimulating factor 1 | CSF-1 | P09603 |
| 101 | Macrophage receptor MARCO | MARCO | Q9UEW3 |
| 102 | Matrix metalloproteinase-1 | MMP-1 | P03956 |
| 103 | Matrix metalloproteinase-10 | MMP-10 | P09238 |
| 104 | Matrix metalloproteinase-12 | MMP-12 | P39900 |
| 105 | Matrix metalloproteinase-7 | MMP-7 | P09237 |
| 106 | Melusin | ITGB1BP2 | Q9UKP3 |
| 107 | Monocyte chemotactic protein 1 | MCP-1 | P13500 |
| 108 | Monocyte chemotactic protein 2 | MCP-2 | P80075 |
| 109 | Monocyte chemotactic protein 3 | MCP-3 | P80098 |
| 110 | Monocyte chemotactic protein 4 | MCP-4 | Q99616 |
| 111 | Natriuretic peptides B | BNP | P16860 |
| 112 | Natural killer cell receptor 2B4 | CD244 | Q9BZW8 |
| 113 | Neurotrophin-3 | NT-3 | P20783 |
| 114 | Neurturin | NRTN | Q99748 |
| 115 | NF-kappa-B essential modulator | NEMO | Q9Y6K9 |
| 116 | Oncostatin-M | OSM | P13725 |
| 117 | Osteoclast-associated immunoglobulin-like receptor | hOSCAR | Q8IYS5 |
| 118 | Osteoprotegerin | OPG | O00300 |
| 119 | Pappalysin-1 | PAPPA | Q13219 |
| 120 | Pentraxin-related protein PTX3 | PTX3 | P26022 |
| 121 | Placenta growth factor | PGF | P49763 |
| 122 | Platelet-derived growth factor subunit B | PDGF subunit B | P01127 |
| 123 | Poly [ADP-ribose] polymerase 1 | PARP-1 | P09874 |
| 124 | Polymeric immunoglobulin receptor | PIgR | P01833 |
| 125 | Programmed cell death 1 ligand 1 | PD-L1 | Q9NZQ7 |
| 126 | Programmed cell death 1 ligand 2 | PD-L2 | Q9BQ51 |
| 127 | Proheparin-binding EGF-like growth factor | HB-EGF | Q99075 |
| 128 | Pro-interleukin-16 | IL16 | Q14005 |
| 129 | Prolargin | PRELP | P51888 |
| 130 | Prostasin | PRSS8 | Q16651 |
| 131 | Protein AMBP | AMBP | P02760 |
| 132 | Protein S100-A12 | EN-RAGE | P80511 |
| 133 | Proteinase-activated receptor 1 | PAR-1 | P25116 |
| 134 | Protein-glutamine gamma-glutamyltransferase 2 | TGM2 | P21980 |
| 135 | Proto-oncogene tyrosine-protein kinase Src | SRC | P12931 |
| 136 | P-selectin glycoprotein ligand 1 | PSGL-1 | Q14242 |
| 137 | Receptor for advanced glycosylation end products | RAGE | Q15109 |
| 138 | Renin | REN | P00797 |
| 139 | Serine protease 27 | PRSS27 | Q9BQR3 |
| 140 | Serine/threonine-protein kinase 4 | STK4 | Q13043 |
| 141 | Serpin A12 | SERPINA12 | Q8IW75 |
| 142 | Signaling lymphocytic activation molecule | SLAMF1 | Q13291 |
| 143 | SIR2-like protein 2 | SIRT2 | Q8IXJ6 |
| 144 | SLAM family member 5 | CD84 | Q9UIB8 |
| 145 | SLAM family member 7 | SLAMF7 | Q9NQ25 |
| 146 | Sortilin | SORT1 | Q99523 |
| 147 | Spondin-2 | SPON2 | Q9BUD6 |
| 148 | STAM-binding protein | STAMBP | O95630 |
| 149 | Stem cell factor | SCF | P21583 |
| 150 | Sulfotransferase 1A1 | ST1A1 | P50225 |
| 151 | Superoxide dismutase [Mn], mitochondrial | SOD2 | P04179 |
| 152 | T cell surface glycoprotein CD6 isoform | CD6 | Q8WWJ7 |
| 153 | T-cell surface glycoprotein CD4 | CD4 | P01730 |
| 154 | T-cell surface glycoprotein CD5 | CD5 | P06127 |
| 155 | T-cell surface glycoprotein CD8 alpha chain | CD8A | P01732 |
| 156 | Thrombomodulin | TM | P07204 |
| 157 | Thrombopoietin | THPO | P40225 |
| 158 | Thrombospondin-2 | THBS2 | P35442 |
| 159 | Thymic stromal lymphopoietin | TSLP | Q969D9 |
| 160 | Tissue factor | TF | P13726 |
| 161 | TNF receptor superfamily member 10A | TNFRSF10A | O00220 |
| 162 | TNF receptor superfamily member 11A | TNFRSF11A | Q9Y6Q6 |
| 163 | TNF receptor superfamily member 13B | TNFRSF13B | O14836 |
| 164 | TNF-beta | TNFB | P01374 |
| 165 | TNF-related activation-induced cytokine | TRANCE | O14788 |
| 166 | TNF-related apoptosis-inducing ligand | TRAIL | P50591 |
| 167 | TNF-related apoptosis-inducing ligand receptor 2 | TRAIL-R2 | O14763 |
| 168 | Transforming growth factor alpha | TGF-alpha | P01135 |
| 169 | Tumor necrosis factor | TNF | P01375 |
| 170 | Tumor necrosis factor (Ligand) superfamily, member 12 | TWEAK | O43508 |
| 171 | Tumor necrosis factor ligand superfamily member 14 | TNFSF14 | O43557 |
| 172 | Tumor necrosis factor receptor superfamily member 9 | TNFRSF9 | Q07011 |
| 173 | Tyrosine-protein kinase Mer | MERTK | Q12866 |
| 174 | Urokinase-type plasminogen activator | uPA | P00749 |
| 175 | Vascular endothelial growth factor A | VEGF-A | P15692 |
| 176 | Vascular endothelial growth factor D | VEGFD | O43915 |
| 177 | V-set and immunoglobulin domain-containing protein 2 | VSIG2 | Q96IQ7 |

# Table S2. Outcomes at 18 months follow-up

| EVENT | N=229 |
| --- | --- |
| Composite endpoint (MACE) | 36 (16) |
| Acute cardiovascular event, n (%)  STEMI, n (%)  NSTEMI, n (%)  Unstable angina, n (%)  Stroke, n (%)  Transient ischemic attack, n (%)  Acute limb ischemia, n (%) | 31 (14)  4 (2)  1 (0)  23 (10)  3 (1)  2 (1)  4 (2) |
| Coronary revascularization, n (%)  Acute setting, n (%)  Symptomatic relief, n (%) | 26 (11)  8 (3)  18 (8) |
| Cardiovascular death, n (%) | 8 (3) |
| All-cause death, n (%) | 11 (5) |

# MACE = major adverse cardiovascular events; NSTEMI = non-ST elevation myocardial infarction; STEMI = ST-segment elevation myocardial infarction.

**Table S3.** Baseline characteristics with hazard ratios for major adverse cardiovascular events

| **PARAMETER** | **HR** | **95% Confidence Intervals** | ***p*-value** |
| --- | --- | --- | --- |
| **CLINICAL DATA** |  |  |  |
| Age (years) | 1.048 | 1.008-1.090 | 0.018 |
| Male sex | 1.123 | 0.555-2.453 | 0.757 |
| BMI (kg/m2) | 1.048 | 0.949-1.151 | 0.344 |
| Smokers | 0.544 | 0.088-1.791 | 0.403 |
| Heart rate (beats/min) | 1.006 | 0.974-1.035 | 0.676 |
| Hypertension | 1.191 | 0.426-4.956 | 0.772 |
| Diabetes | 1.478 | 0.743-2.875 | 0.259 |
| **LABORATORY DATA** |  |  |  |
| Total cholesterol (mg/dl) | 1.006 | 0.999-1.012 | 0.053 |
| LDL-cholesterol (mg/dl) | 1.008 | 0.999-1.015 | 0.049 |
| HDL-cholesterol (mg/dl) | 0.985 | 0.955-1.013 | 0.316 |
| Creatinine (mg/dl) | 1.568 | 0.842-2.390 | 0.080 |
| eGFR (ml/min/1.73m^2^) | 0.984 | 0.971-0.997 | 0.022 |
| **ECHOCARDIOGRAPHIC DATA** |  |  |  |
| LVEF (%) | 0.974 | 0.944-1.009 | 0.126 |
| **CONCOMITANT MEDICATION** |  |  |  |
| Aspirin | 0.674 | 0.320-1.594 | 0.329 |
| P2Y12 inhibitor | 1.992 | 0.968-4.501 | 0.074 |
| Betablocker | 0.454 | 0.227-0.965 | 0.030 |
| ACE inhibitor/ARB | 0.770 | 0.380-1.683 | 0.485 |
| Calcium channel blocker | 0.925 | 0.423-1.869 | 0.835 |
| Nitrate | 1.480 | 0.744-2.879 | 0.251 |
| Statin | 0.651 | 0.261-2.075 | 0.408 |
| Fibrate | 1.205 | 0.289-3.375 | 0.757 |
| **ATHEROSCLEROSIS SEVERITY** |  |  |  |
| SYNTAX Score I | 1.019 | 0.985-1.049 | 0.229 |
| SYNTAX Score II – PCI | 1.042 | 1.008-1.076 | 0.013 |
| SYNTAX Score II – CABG | 1.034 | 0.998-1.068 | 0.054 |
| Complete revascularization | 0.868 | 0.397-1.756 | 0.707 |

ACE = angiotensin converting enzyme; ARB = angiotensin receptor blocker; BMI = body mass index; CABG = coronary artery bypass graft; CD = cluster of differentiation; eGFR = estimated glomerular filtration rate; HDL-cholesterol = high density lipoprotein cholesterol; LDL-cholesterol = low density lipoprotein cholesterol; m = mean; mil = million; n = number; PBMCs = peripheral blood mononuclear cells; PCI = percutaneous coronary intervention; Q1 = 1st quartile; Q3 = 3rd quartile; WBC = white blood cells.

# Table S4. Plasma proteins with hazard ratios for major adverse cardiovascular events

| BIOMARKER | HR | 95% Confidence Intervals | *p*-value |
| --- | --- | --- | --- |
| TNFRSF13B | 1.653 | 1.301-2.101 | <0.001 |
| CCL3 | 1.566 | 1.234-1.987 | <0.001 |
| DCN | 1.649 | 1.257-2.164 | <0.001 |
| FGF-23 | 1.565 | 1.227-1.997 | <0.001 |
| TRAIL-R2 | 1.613 | 1.233-2.110 | <0.001 |
| TNFRSF10A | 1.691 | 1.251-2.286 | <0.001 |
| FGF-21 | 1.753 | 1.254-2.451 | 0.001 |
| TNFRSF9 | 1.577 | 1.196-2.079 | 0.001 |
| ADM | 1.772 | 1.252-2.507 | 0.001 |
| SPON2 | 1.682 | 1.216-2.328 | 0.002 |
| CXCL10 | 1.558 | 1.180-2.057 | 0.002 |
| SLAMF7 | 1.594 | 1.189-2.136 | 0.002 |
| PRELP | 1.618 | 1.191-2.198 | 0.002 |
| PGF | 1.511 | 1.153-1.980 | 0.003 |
| CTSL1 | 1.559 | 1.161-2.094 | 0.003 |
| TNFRSF11A | 1.538 | 1.155-2.048 | 0.003 |
| IL-10RB | 1.57 | 1.153-2.138 | 0.004 |
| MMP7 | 1.557 | 1.144-2.119 | 0.005 |
| MERTK | 1.485 | 1.126-1.959 | 0.005 |
| LIF-R | 1.629 | 1.152-2.304 | 0.006 |
| CXCL9 | 1.508 | 1.125-2.022 | 0.006 |
| Gal-9 | 1.492 | 1.120-1.988 | 0.006 |
| CX3CL1 | 1.528 | 1.123-2.079 | 0.007 |
| CD4 | 1.412 | 1.096-1.819 | 0.008 |
| SLAMF1 | 1.542 | 1.121-2.122 | 0.008 |
| MMP12 | 1.51 | 1.109-2.056 | 0.009 |
| KIM1 | 1.461 | 1.086-1.966 | 0.012 |
| REN | 1.657 | 1.109-2.474 | 0.014 |
| CST-5 | 1.508 | 1.088-2.089 | 0.014 |
| PRSS8 | 1.529 | 1.076-2.173 | 0.018 |
| VEGFA | 1.355 | 1.045-1.756 | 0.022 |
| OPG | 1.456 | 1.049-2.021 | 0.025 |
| CD40 | 1.352 | 1.038-1.760 | 0.025 |
| IL-8 | 1.352 | 1.035-1.768 | 0.027 |
| PD-L1 | 1.389 | 1.036-1.862 | 0.028 |
| CCL23 | 1.447 | 1.037-2.020 | 0.03 |
| AMBP | 1.438 | 1.029-2.009 | 0.033 |
| PlgR | 1.386 | 1.024-1.876 | 0.034 |
| TF | 1.418 | 1.024-1.965 | 0.036 |
| TGF-alpha | 1.334 | 1.019-1.747 | 0.036 |
| CD5 | 1.311 | 1.017-1.690 | 0.036 |
| IL-2RB | 1.334 | 1.017-1.750 | 0.037 |
| TNF | 1.388 | 1.020-1.888 | 0.037 |
| LEP | 1.566 | 1.020-2.404 | 0.041 |
| CCL25 | 1.42 | 1.012-1.993 | 0.042 |
| SORT1 | 1.305 | 1.008-1.688 | 0.043 |
| RAGE | 1.384 | 1.004-1.908 | 0.047 |
| FABP2 | 1.426 | 1.003-2.029 | 0.048 |
| TM | 1.375 | 0.998-1.893 | 0.051 |
| IL-15RA | 1.374 | 0.999-1.891 | 0.051 |
| HGF | 1.287 | 0.995-1.665 | 0.055 |
| IL-4 | 0.787 | 0.615-1.008 | 0.057 |
| IL-17C | 1.347 | 0.983-1.846 | 0.064 |
| LPL | 1.397 | 0.980-1.992 | 0.065 |
| XCL1 | 1.32 | 0.979-1.780 | 0.069 |
| IL-18 | 1.334 | 0.971-1.833 | 0.075 |
| IL-20RA | 1.285 | 0.973-1.697 | 0.077 |
| FGF-5 | 1.297 | 0.971-1.731 | 0.078 |
| MCP-1 | 1.273 | 0.970-1.671 | 0.082 |
| THPO | 1.305 | 0.965-1.766 | 0.084 |
| EN-RAGE | 1.281 | 0.960-1.708 | 0.092 |
| IL-10 | 1.272 | 0.958-1.687 | 0.096 |
| VEGFD | 2.146 | 0.856-5.380 | 0.104 |
| CSF-1 | 1.3 | 0.935-1.807 | 0.119 |
| VSIG2 | 1.305 | 0.930-1.831 | 0.124 |
| NRTN | 1.244 | 0.938-1.648 | 0.13 |
| AGRP | 1.264 | 0.925-1.728 | 0.142 |
| IL-17A | 1.274 | 0.921-1.763 | 0.143 |
| Flt3l | 1.294 | 0.917-1.825 | 0.143 |
| GDNF | 1.279 | 0.913-1.791 | 0.152 |
| GH | 1.276 | 0.910-1.788 | 0.158 |
| Beta-NGF | 1.191 | 0.934-1.520 | 0.159 |
| PDGF subunit B | 0.793 | 0.572-1.099 | 0.164 |
| PD-L2 | 1.248 | 0.888-1.753 | 0.202 |
| GT | 1.242 | 0.889-1.735 | 0.204 |
| IL-1RA | 1.226 | 0.894-1.682 | 0.207 |
| LOX-1 | 1.198 | 0.905-1.586 | 0.207 |
| IL-6 | 1.239 | 0.888-1.729 | 0.207 |
| HAOX1 | 0.818 | 0.593-1.128 | 0.221 |
| hOSCAR | 1.233 | 0.880-1.727 | 0.225 |
| IL-4RA | 1.206 | 0.885-1.643 | 0.236 |
| ADAM-TS13 | 1.164 | 0.904-1.497 | 0.239 |
| GIF | 0.877 | 0.700-1.099 | 0.253 |
| FGF-19 | 1.209 | 0.869-1.684 | 0.26 |
| IL-27 | 1.217 | 0.858-1.727 | 0.27 |
| IL-12B | 1.204 | 0.865-1.676 | 0.272 |
| ACE2 | 1.189 | 0.870-1.624 | 0.279 |
| CCL20 | 1.186 | 0.863-1.630 | 0.294 |
| IgG Fc RECEPTOR II-b | 1.204 | 0.843-1.719 | 0.306 |
| TIE2 | 1.184 | 0.856-1.639 | 0.307 |
| IDUA | 1.194 | 0.849-1.680 | 0.309 |
| IL-17D | 1.155 | 0.873-1.528 | 0.314 |
| CD244 | 1.169 | 0.863-1.584 | 0.314 |
| TRANCE | 1.189 | 0.847-1.670 | 0.318 |
| LAP TGF-beta-1 | 1.162 | 0.863-1.567 | 0.323 |
| LIF | 1.17 | 0.854-1.603 | 0.329 |
| PARP-1 | 1.142 | 0.867-1.504 | 0.345 |
| PAR-1 | 1.199 | 0.821-1.750 | 0.347 |
| ANGPT1 | 0.855 | 0.611-1.195 | 0.358 |
| CA5A | 1.164 | 0.839-1.615 | 0.363 |
| MCP-3 | 1.161 | 0.836-1.611 | 0.373 |
| ITGB1BP2 | 0.856 | 0.600-1.221 | 0.392 |
| IL1RL2 | 0.877 | 0.648-1.187 | 0.395 |
| IL-16 | 1.139 | 0.843-1.540 | 0.397 |
| FS | 1.156 | 0.825-1.621 | 0.4 |
| PRSS27 | 1.151 | 0.827-1.602 | 0.404 |
| CEACAM8 | 1.134 | 0.842-1.527 | 0.409 |
| 4E-BP1 | 1.141 | 0.831-1.568 | 0.415 |
| CXCL1 | 1.133 | 0.836-1.536 | 0.421 |
| SERPINA12 | 1.139 | 0.826-1.572 | 0.427 |
| MMP-1 | 0.88 | 0.633-1.225 | 0.448 |
| TNFSF14 | 1.12 | 0.835-1.502 | 0.45 |
| BNP | 1.143 | 0.806-1.621 | 0.453 |
| uPA | 1.128 | 0.822-1.548 | 0.457 |
| MCP-2 | 1.134 | 0.809-1.589 | 0.465 |
| IL-20 | 1.136 | 0.806-1.600 | 0.466 |
| IL-2 | 1.136 | 0.805-1.604 | 0.467 |
| SCF | 1.144 | 0.795-1.645 | 0.469 |
| CD84 | 1.114 | 0.826-1.502 | 0.48 |
| TSLP | 0.898 | 0.663-1.216 | 0.486 |
| DNER | 0.899 | 0.667-1.212 | 0.486 |
| CCL17 | 1.117 | 0.814-1.532 | 0.492 |
| THBS2 | 1.12 | 0.807-1.554 | 0.5 |
| CCL19 | 0.89 | 0.635-1.248 | 0.5 |
| BMP-6 | 0.895 | 0.646-1.240 | 0.505 |
| CXCL11 | 0.903 | 0.638-1.277 | 0.562 |
| CCL4 | 1.094 | 0.800-1.498 | 0.573 |
| PSGL-1 | 1.105 | 0.775-1.576 | 0.581 |
| BOC | 1.092 | 0.785-1.517 | 0.602 |
| GDF-2 | 0.919 | 0.665-1.271 | 0.61 |
| Dkk-1 | 1.084 | 0.792-1.484 | 0.613 |
| IL-33 | 1.085 | 0.780-1.509 | 0.627 |
| CDCP1 | 1.084 | 0.777-1.512 | 0.635 |
| TWEAK | 1.074 | 0.799-1.444 | 0.637 |
| TGM2 | 0.931 | 0.691-1.254 | 0.638 |
| MARCO | 1.081 | 0.780-1.500 | 0.639 |
| MMP-10 | 1.08 | 0.779-1.496 | 0.645 |
| TNFB | 1.082 | 0.770-1.520 | 0.649 |
| CXCL6 | 1.071 | 0.779-1.473 | 0.671 |
| IL-7 | 1.07 | 0.782-1.462 | 0.673 |
| AXIN1 | 0.934 | 0.667-1.308 | 0.691 |
| ADA | 0.934 | 0.656-1.331 | 0.706 |
| IL-5 | 0.941 | 0.683-1.298 | 0.712 |
| CASP8 | 1.055 | 0.783-1.422 | 0.723 |
| PAPPA | 1.064 | 0.755-1.498 | 0.724 |
| IFN-gamma | 1.057 | 0.764-1.462 | 0.738 |
| TRAIL | 1.055 | 0.759-1.465 | 0.751 |
| ST1A1 | 0.947 | 0.678-1.323 | 0.751 |
| SIRT2 | 1.049 | 0.767-1.434 | 0.765 |
| ARTN | 0.951 | 0.676-1.339 | 0.773 |
| IL-10RA | 1.042 | 0.763-1.423 | 0.795 |
| IL-22RA1 | 0.961 | 0.692-1.333 | 0.81 |
| IL-18R1 | 0.964 | 0.692-1.343 | 0.829 |
| IL-24 | 0.966 | 0.693-1.346 | 0.838 |
| CXCL5 | 1.033 | 0.748-1.428 | 0.842 |
| CD6 | 1.033 | 0.745-1.432 | 0.845 |
| HB-EGF | 1.031 | 0.753-1.411 | 0.849 |
| PTX3 | 0.971 | 0.694-1.358 | 0.864 |
| NEMO | 1.027 | 0.752-1.402 | 0.867 |
| SRC | 1.026 | 0.746-1.411 | 0.874 |
| IL-13 | 1.023 | 0.742-1.412 | 0.889 |
| MCP-4 | 1.022 | 0.740-1.413 | 0.894 |
| STAMBP | 0.979 | 0.708-1.355 | 0.9 |
| GLO1 | 1.021 | 0.740-1.407 | 0.901 |
| HSP27 | 1.021 | 0.740-1.409 | 0.901 |
| STK4 | 0.984 | 0.713-1.358 | 0.922 |
| CCL11 | 0.985 | 0.715-1.356 | 0.924 |
| CCL28 | 1.015 | 0.739-1.396 | 0.925 |
| HO-1 | 0.986 | 0.713-1.362 | 0.93 |
| OSM | 0.986 | 0.716-1.359 | 0.932 |
| DECR1 | 1.012 | 0.736-1.393 | 0.939 |
| SOD2 | 0.989 | 0.706-1.384 | 0.948 |
| CTRC | 0.989 | 0.708-1.382 | 0.948 |
| NT-3 | 0.992 | 0.715-1.376 | 0.962 |
| CD8A | 0.994 | 0.711-1.389 | 0.971 |
| CD40-L | 1.001 | 0.727-1.379 | 0.994 |

# Hazard ratios represent risk per 1 SD increase in each biomarker. HR = hazard ratio
